# A Genetic Variant in 12q13, a Possible Risk Factor for Bipolar Disorder, Is Associated with Depressive State, Accounting for Stressful Life Events

**DOI:** 10.1371/journal.pone.0115135

**Published:** 2014-12-17

**Authors:** Ayu Shimasaki, Kenji Kondo, Takeo Saito, Kosei Esaki, Yasuyo Otsuka, Keiko Mano, Masashi Ikeda, Nakao Iwata

**Affiliations:** 1 Department of Psychiatry, Fujita Health University School of Medicine, Toyoake, Aichi, Japan; 2 Division of Nursing, Fujita Health University Hospital, Toyoake, Aichi, Japan; UTHSCSH, United States of America

## Abstract

Genome-wide association studies (GWASs) have identified a number of susceptibility genes for schizophrenia (SCZ) and bipolar disorder (BD). However, the identification of risk genes for major depressive disorder (MDD) has been unsuccessful because the etiology of MDD is more influenced by environmental factors; thus, gene–environment (G×E) interactions are important, such as interplay with stressful life events (SLEs). We assessed the G×E interactions and main effects of genes targeting depressive symptoms. Using a case–control design, 922 hospital staff members were evaluated for depressive symptoms according to Beck Depressive Inventory (BDI; “depression” and “control” groups were classified by scores of 10 in the BDI test), SLEs, and personality. A total of sixty-three genetic variants were selected on the basis of previous GWASs of MDD, SCZ, and BD as well as candidate-gene (*SLC6A4*, *BDNF, DBH*, and *FKBP5*) studies. Logistic regression analysis revealed a marginally significant interaction (genetic variant × SLE) at rs4523957 (P_uncorrected_ = 0.0034) with depression and a significant association of single nucleotide polymorphism identified from evidence of BD GWAS (rs7296288, downstream of *DHH* at 12q13.1) with depression as the main effect (P_uncorrected_ = 9.4×10^−4^, P_corrected_ = 0.0424). We also found that SLEs had a larger impact on depression (odds ratio∼3), as reported previously. These results suggest that *DHH* plays a possible role in depression etiology; however, variants from MDD or SCZ GWAS evidence or candidate genes showed no significant associations or minimal effects of interactions with SLEs on depression.

## Introduction

Major depressive disorder (MDD) is a common and debilitating disorder with pervasive impact on the quality of life of patients and their families. Approximately 15% of the general population suffers from this condition throughout their life spans [1]. Several lines of evidence from epidemiological studies suggest that the heritability of MDD is significant but modest (approximately 40%) [2]; thus, numerous genetic association studies on candidate genes and, more recently, genome-wide association studies (GWASs) have been conducted. However, no risk gene for MDD [3] has been identified till date, although GWASs for other psychiatric disorders such as schizophrenia (SCZ) [4] and bipolar disorder (BD) [5] have identified a number of risk genes.

One possible reason for the failure to identify MDD-related genes has been suggested from the phenotyping of MDD. Despite the development of standardized diagnostic manuals for general psychiatry, such as the Diagnostic and Statistical Manual of Mental Disorders (DSM) and International Statistical Classification of Diseases and Related Health Problems (ICD), the diagnostic reliability for MDD is insufficient [6]. In addition, together with the high prevalence of MDD, the definition of “healthy controls” as comparative subjects is difficult in case–control studies; even if the “controls” have not been diagnosed for MDD at the time of enrollment, the high prevalence of MDD does not ensure the future development of MDD. Therefore, a very large number of samples is required to compensate for the loss of statistical power. However, even in mega-analysis of GWASs, in which the largest samples were analyzed, no risk of single nucleotide polymorphism (SNP) with genome-wide significance has been identified [3].

Another concern that should be considered involves the environmental or clinical factors. Among these, the promising candidates are sex (male/female), stressful life events (SLEs), personality traits (specifically neuroticism), and age. In particular, SLEs have the potential for interactions with genetic components; most studies have focused on promoter polymorphism (5-HTTLPR) in the serotonin transporter gene (*SLC6A4*) or Val66Met polymorphism in brain-derived neurotrophic factor (*BDNF*) [7], [8]. Although multiple meta-analyses of gene–environment (G×E) interactions between 5-HTTLPR and SLEs have not shown significant associations with MDD [9], [10], [11], little is known about other genes that may interact with SLEs. Furthermore, even if there is no G×E interaction for MDD, SLEs should be taken into account for accurately evaluating the SNP risk as a main effect on the complex phenotype, such as MDD.

The aim of this study was to examine the associations between depressive symptoms, environmental/clinical factors, and genetic variants. In addition to SNPs or variants of candidate genes identified from GWASs of MDD, we selected SNPs on the basis of previous GWASs of SCZ and BD because risk SNPs have pleiotropic effects on psychiatric disorders [12]. All subjects used in this study were nurses working in a university hospital who had a higher risk for developing MDD than the general population, probably because their work involves a high-degree of responsibility and requires high motivation and long work periods, including night shifts.

## Materials and Methods

### Ethics statements

Written informed consent was obtained from each subject after the procedures had been fully explained. This study was performed in accordance with the World Medical Association's Declaration of Helsinki and approved by the ethics committees at Fujita Health University.

### Subjects

All participants were nurses working at Fujita Health University Hospital, and were self-identified as members of the Japanese population. We approached 1296 subjects, and a total of 926 (response rate, 71.4%: 90 males and 836 females; mean age, 29.3 years; range, 20–62 years) agreed to participate. However, four participants were excluded due to insufficient answer for the following questionnaire.

The samples were collected in two phases: April 2012 (835 subjects) and April 2013 (87 subjects). All subjects were evaluated for (1) depressive symptoms according to the Beck Depressive Inventory I (BDI; a 20-item questionnaire) [13]; (2) SLEs according to the List of Threatening Events (LTE) Questionnaire [14] (12 life events within 6 months that were found to have long-term negative effects on most people); and (3) personality traits according to the Neuroticism–Extraversion–Openness Five-Factor Inventory [15] (NEO-FFI; 60 items assessing five personality traits, including neuroticism, extraversion, openness to experience, agreeableness, and conscientiousness).

### SNP selection, genotyping, and quality control

We listed a total of 63 SNPs/variants in this study (S1 Table). Fifty-nine SNPs were selected from GWAS data published prior to December 2013, and the following selection criteria were used: the P value must have been 1) <1×10^−4^ in mega-analysis of GWASs for MDD [3] (18 SNPs); 2) <1×10^−7^ in mega-analysis of GWAS (based on the results of the primary or the combined results) for BD [5] (five SNPs); 3) <1×10^−7^ in mega-analysis of GWASs based on the primary results (SNPs in linkage equilibrium defined by PGC or SNPs in non-MHC region were included) or in those based on the combined results for SCZ [4] (32 SNPs); and 4) <5×10^−8^ in mega-analysis of GWASs for five major psychiatric disorders (four SNPs; however, one SNP, rs1024582, was excluded because of a known LD, with other *CACNA1C* SNPs. Thus, three SNPs were included) [12]. Another three SNPs and one variant (5-HTTLPR) were selected on the basis of candidate gene (*SLC6A4* [9], [10], [11], *BDNF* [8], *FKBP5* [16], [17], and *DBH*) studies, which were prioritized as risk genes for MDD in score-based analysis [18], with significant P values (P<0.05) detected in the PGC-MDD database [3]. We also confirmed that all SNPs had minor allele frequencies (MAFs) >1% according to the HapMapJPT panel (phase II or III: MAFs of six SNPs were zero and thus excluded).

The Oragene DNA Self-Collection Kit (DNAgenotek, Ontario, Canada) was used to collect and extract DNA from the saliva of 922 subjects (89 males and 833 females). We genotyped these SNPs using the Sequenom iPLEX Gold System (Sequenom, San Diego, CA) according to the manufacturer's protocol. Genotyping call performed by using the Typer 4.0 program (Sequenom, San Diego, CA). Another possible risk variant, 5-HTTLPR, was genotyped by the TaqMan assay (Life Technologies, Grand Island, NY) in the manner described by Hu et al [19]. Ninety-six samples were genotyped according to the classical PCR method, and the concordance rate between the TaqMan and PCR methods was 100%.

In the primer design step, one SNP (rs171748) was excluded. Moreover, in the optimization step, because of the unacceptable genotyping clustering by visual inspection, four SNPs (rs114002140, rs2183696, rs11062146 and rs11532322) were excluded. For quality control, we removed samples and SNPs with call rates <90% (N of samples  = 34, N of SNP  = 0). In addition, SNPs with Hardy-Weinberg Equilibrium (<0.001: rs393093) and low MAF (<0.01: rs7746199) were removed. Lastly, four SNPs (rs4687552, rs113113059, rs11191454 and rs4765913) had proxy SNPs; these were LD pruned using the criterion of r^2^<0.25 in our genotyping data. In total, we analyzed 888 samples and 45 SNPs used in association analysis.

### Statistical analysis

The chi-square test was used to test the Hardy–Weinberg equilibrium for genotype distribution. To compare the clinical backgrounds, Student's t test for continuous values (BDI score, age and employment period [20]), the chi-square test for sex, and the Mann–Whitney U test for SLEs were used. The correlation between depressive symptoms measured by BDI and personality traits assessed by NEO-FFI was calculated using the Pearson's method.

To examine the association of the genetic variants, SLEs, and their interactions with the depressive symptoms assessed by BDI, multiple logistic regression analysis was performed for examining the main effects and two-way interactions. The dependent variable was the categorical phenotype for the depressive state (depressive subjects: BDI ≥10; controls: BDI <10; 297 subjects were assigned to cases, and the rest were designated as nondepressive subjects) because a previous study indicated that this threshold could reflect the depressive state [21]. In this study, approximately 33% of the participants were regarded as being in a depressive state, and this proportion was similar to that observed in another study targeting nurses [20]. Genetic variants (additive model), SLEs (0 or >1), and their interactions (genetic variants × SLE) were entered as independent variables with covariation of sex and age.

In terms of personality traits, neuroticism particularly showed a very high correlation with the BDI score; therefore, we excluded the personality traits as covariates in this model. The significance level was set to P<0.05 (two-tailed). All statistical analyses were performed by using R or PLINK ver1.07 software [22].

## Results

### Clinical backgrounds

The clinical backgrounds of all depressive and nondepressive subjects are shown in Table 1. No significant differences in the distributions of age, sex and employment period were observed between the two groups, except in the scores for neuroticism and number of SLEs.

**Table 1:** Clinical backgrounds of the subjects with depressive state and non-depressive state.

factors	total	depressive state (BDI ≥10)	non-depressive state (BDI <10)	P-value
gender (male/female)	89/833	30/279	59/554	0.968
BDI*	8.4±7.3	16.8±5.9	4.1±2.9	<0.0001
Age	29.8±8.4	29.2±7.9	30.0±8.6	0.149
neuroticism	55.2±10.7	63.3±8.3	51.1±9.3	<0.0001
Employment period (years)	7.8±8.1	7.2±7.7	8.1±8.3	0.117
SLE**	0.38±0.74	0.61±0.92	0.20±0.40	<0.0001

Numbers represent means ± SDs.

*BDI: Beck Depressive Inventory, **SLE: Stressful life event.

Initial logistic regression analysis (independent variables: age, sex, SLE, and neuroticism) revealed that neuroticism showed a highly significant association with the depressive state (S2 Table). Therefore, we examined the correlation between BDI score and personality traits and found a significant correlation with neuroticism (Pearson's r^2^ = 0.60, P = 2.2×10^−16^). When examining the hypothesis that effects of SNP or interactions between SNP and SLEs are associated with depressive state, co-linearity is a major problem; therefore, we did not include neuroticism in the following regression model.

### Main effect of SNP and SNP × SLE interaction on the depressive state

Application of the quality control procedure showed that 45 SNPs and 888 subjects were eligible for examination of the main effect of SNP and SNP × SLE interactions on the depressive state. In the logistic regression analysis, SLEs (experienced or not experienced) were associated with the depressive state for all SNPs (P = 0.0042−4.0×10^−11^). The effect size was relatively large, which indicated that the presence of SLEs has a high impact on developing the depressive state. Three SNPs showed significant associations as main effects on the depressive state (Table 2; full results are shown in S3 Table). The top finding was observed at rs7296288, which was selected on the basis of the BD GWAS results. Even after correction for multiple testing (Bonferroni), the P value of this SNP remained significant (P_corrected_ = 0.0424).

**Table 2:** Significant association of SNPs as main effects and G×E interactions on depressive state.

Chr^a^	SNP^b^	BP^c^	gene^d^	A1^e^	A2^f^	F A^g^	F U^h^	model^i^	P_uncorrected_	OR^k^
6	rs1360780	35607571	FKBP5	T	C	0.286	0.240	ADD	0.0255	1.36
								ADDxSLE	0.251	0.75
9	rs1108580	136505114	DBH	G	A	0.148	0.111	ADD	0.0318	1.48
								ADDxSLE	0.472	0.79
12	rs7296288	49479968	DHH	C	A	0.379	0.320	ADD	**0.000943** j	1.56
								ADDxSLE	0.0197	0.58
17	rs4523957	2208899	SMG6	G	T	0.281	0.221	ADD	0.462	1.11
								ADDxSLE	0.00337	2.24

a Chr: chromosome,

b SNP: single nucleotide polymorphism,

c BP: base position,

d Closest gene ±100 kb,

e A1: minor allele based on total sample,

f A2: alternative allele,

g F A: minor allele frequency in depressive state,

h F U: minor allele frequency in non-depressive state,

i ADD: additive model, SLE: stressful life event,

j Bold numbers represents significant P values after Bonferroni correction,

k OR: odds ratio.

The SNP × SLE interaction showed a marginally significant association (P<0.05) only at two loci (rs4523957 and rs7296288), one of which was identical to the result for the main effect of SNP (rs7296288; Table 2; the full results are shown in S2 Table). Notably, other candidate variants, including *5-HTTLPR*, *BDNF*, and *FKBP5* were not associated with the depressive state in our sample.

## Discussion

In this study, we determined whether the genetic variants selected on the basis of previous GWASs of MDD, BD, and SCZ or classical candidate genes were associated with the depressive state and assumed that there were genetic variant × SLE interactions (G×E interactions). All our subjects were nurses who worked at a university hospital; therefore, these subjects did not reflect the risk for depressive symptoms of the general population. However, because working as a nurse is a risk factor for the development of MDD owing to hard work, the need to be highly motivated, and the high level of responsibility, our subject sample set was regarded as being at high risk for depression.

In this study, we found that SLEs were risk factors for depressive symptoms and had moderate to high effect magnitudes (odds ratio ∼3). This finding is in line with those of previous studies [23], [24]; therefore, the nurses who experienced SLEs ≤6 months as measured by LTE required more care to prevent the development of depression. A personality trait, neuroticism, was highly correlated with the depressive symptoms evaluated by BDI. This result possibly indicates that neuroticism is a state reflecting depressive symptoms rather than a trait; alternatively, as described previously, neuroticism is a strong risk factor for depression [25], [26]. We cannot determine which possibility is correct; however, this was not the main issue to be examined in this study.

Logistic regression analysis to determine the main effects of SNP and G×E interactions revealed that a possible BD risk SNP, rs7296288, had a significant main effect on depressive symptoms; however, there was a minimal effect of the G×E interaction. A significant SNP, rs7296288 (downstream of *DHH*), was identified in mega-analysis of BD [5]. Because the P value of this SNP in that study [5] was not at a genome-wide significance level but was marginally above this threshold, this gene remains unclear as a risk factor for BD. In addition, the functional relevance of this gene is not clear for the development of BD and/or MDD till date, although this gene plays a role in male gonadal differentiation and perineural development in peripheral nerve systems [27]. However, we consulted the PGC MDD datasets to examine the association between MDD and the controls in the largest sample set as an independent replication for mood disorders. We found a marginal level of significance for this SNP, with the same direction of the effect (P = 0.00334; risk allele, C; odds ratio  = 1.07) [3]. Furthermore, Cross-Disorder Group of the Psychiatric Genomics Consortium revealed a significant association between SNP and psychiatric disorders (P = 1.7×10^−4^; risk allele, C; odds ratio  = 1.05) [12]. Therefore, even without considering the G×E interaction, the LD region with this SNP is a possible risk factor for mood disorders, including MDD and BD, not only for Caucasians but also for the Japanese population. SLEs as covariates may provide sufficient enhancement for extracting the risk as a main effect on MDD.

Classical candidate genes such as *SLC6A4, BDNF, FKBP5*, and *DBH* that have been the subjects of extensive research on depression and may have G×E interactive effects on depression were not associated with the depressive state. Although the sample size in our study was much smaller than those in recent meta-analyses of G×E interactions of *SLC6A4* [9], [10], [11], our results supported the negative trend for an association of the effect of 5-HTTLPR in *SLC6A4*. Although the most recent meta-analysis of *BDNF* [8] showed a significant association of the effect of the G×E interaction on the depression state, as shown in an individual report of *FKBP5* [28], our data could not support these findings. Comparing previous studies and our present study, the main differences were the target subject samples, particularly for *FKBP5*; the main phenotypes in the previous studies were child and/or adolescent depression [16] or post-traumatic stress disorder and related depression [17]. Because we did not evaluate SLEs in childhood, such as child abuse, we could not assess the effects caused by severe SLEs.

A couple of limitations should be noted when interpreting our results. First, the sample size was modest and insufficient for the detection of a small effect size. Power analysis calculated by QUANTO version 1.2.4 software (http://biostats.usc.edu/Quanto.html) suggests that the power of the G×E interaction was insufficient (S4 Table). A larger sample size will be required for future studies. Second, our depressive subjects were those with BDI scores >10, a score that is used as a screening threshold for MDD. This approach implies that the “cases” in our study were not always MDD. However, notably, independent samples from PGC showed a significant association between rs7296288 and MDD.

In summary, these results suggest that *DHH* or genes in LD with rs7296288 play a role in the etiology of depression. However, SNPs selected from MDD/SCZ GWAS evidence or classical candidate genes showed no significant associations. The effect size of G×E interactions between the genetic variants and SLEs for depression were not large, as expected; thus, further studies with larger sample sizes are required.

## Supporting Information

S1 Figure **SNP selection.** BD: Bipolar disorder. MDD: Major depressive disorder. SCZ: Schizophrenia. CDG: Cross-Disorder Group.
(TIF)

S2 Figure **Quality control of selected SNPs.** MAF: minor allele frequency. LD: linkage disequilibrium. HWE: Hardy-Weinberg Equilibrium.
(TIF)

S1 Table **SNP information.** abbreviations: Chr: chromosome, gene: closest gene +/−100 kb, A1: minor allele based on total sample, A2: alternative allele.
(XLSX)

S2 Table **Initial logistic regression analysis (independent variables: age, gender, SLE, and neuroticism).** abbreviations: N: Neuroticism, E: Extraversion, O: Openness, A: Agreeableness, C: Conscientiousness.
(XLSX)

S3 Table **Association analysis of all models including SNPs as main effect and GxE interaction on depressive state.** abbreviations: Chr: chromosome, BP: base position, gene: closest gene +/−100 kb, A1: minor allele based on total sample, A2: alternative allele, NMISS: number of non missing subjects, ADD: additive model, SLE: stressful life event, OR: odds ratio.
(XLSX)

S4 Table **Power analysis.** Assuming: N of subjects: 300 case vs 600 control, significance: 0.05 (two-tailed), mode of inheritance: Log additive, effect size of gene (R_G_): 1.2 and effect size of environment (R_E_): 2. R_GE_: risk of GxE interaction.
(XLSX)
