# Supplementary figures and images for: A Genetic Variant in 12q13, a Possible Risk Factor for Bipolar Disorder, Is Associated with Depressive State, Accounting for Stressful Life Events
Source: PLoS One. 2014 Dec 17;9(12):e115135. doi: 10.1371/journal.pone.0115135 (PMC4269417; doi:10.1371/journal.pone.0115135)

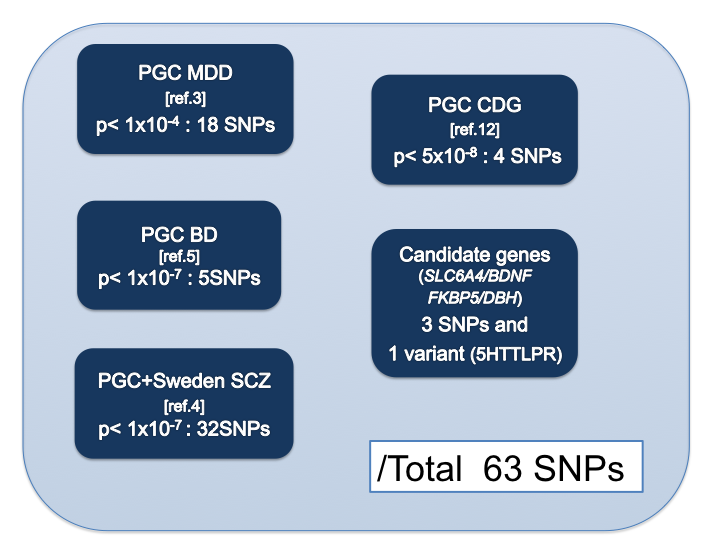

Supplement: S1 Figure — SNP selection. BD: Bipolar disorder. MDD: Major depressive disorder. SCZ: Schizophrenia. CDG: Cross-Disorder Group. (TIF) [file pone.0115135.s001.tif]

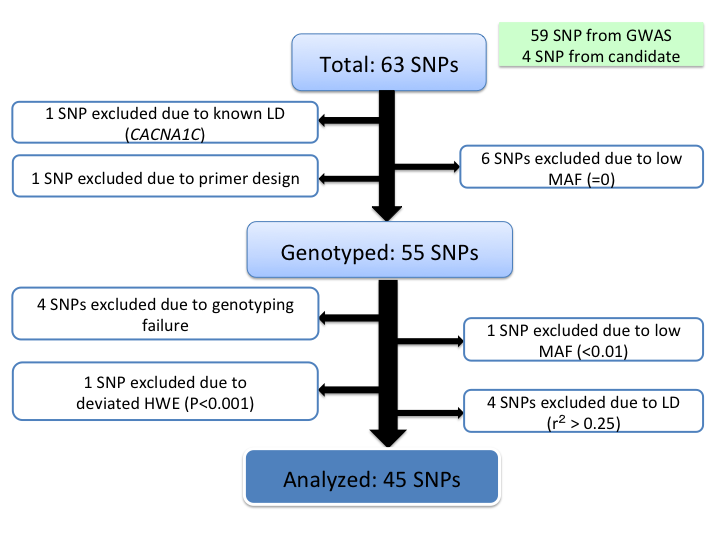

Supplement: S2 Figure — Quality control of selected SNPs. MAF: minor allele frequency. LD: linkage disequilibrium. HWE: Hardy-Weinberg Equilibrium. (TIF) [file pone.0115135.s002.tif]
